# Polymorphism rs1385129 Within Glut1 Gene *SLC2A1* Is Linked to Poor CD4+ T Cell Recovery in Antiretroviral-Treated HIV+ Individuals

**DOI:** 10.3389/fimmu.2018.00900

**Published:** 2018-05-17

**Authors:** Jesse J. R. Masson, Catherine L. Cherry, Nicholas M. Murphy, Isabel Sada-Ovalle, Tabinda Hussain, Riya Palchaudhuri, Jeffrey Martinson, Alan L. Landay, Baki Billah, Suzanne M. Crowe, Clovis S. Palmer

**Affiliations:** ^1^Centre for Biomedical Research, Burnet Institute, Melbourne, VIC, Australia; ^2^Department of Infectious Diseases, Monash University, Melbourne, VIC, Australia; ^3^Faculty of Health Sciences, University of the Witwatersrand, Johannesburg, South Africa; ^4^Monash Institute of Pharmaceutical Sciences, Monash University, Parkville, VIC, Australia; ^5^Preimplantation Genetic Diagnosis, Monash IVF, Melbourne, VIC, Australia; ^6^Unidad de Investigación Instituto Nacional de Enfermedades Respiratorias, Mexico City, Mexico; ^7^Department of Biochemistry and Molecular Biology, Monash Biomedicine Discovery Institute, Monash University, Clayton, VIC, Australia; ^8^Department of Immunology-Microbiology, Rush University Medical Centre, Chicago, IL, United States; ^9^School of Public Health and Preventive Medicine, Monash University, Melbourne, VIC, Australia; ^10^Department of Microbiology and Immunology, University of Melbourne, Melbourne, VIC, Australia

**Keywords:** HIV, CD4+ T cells, immune reconstitution, immunometabolism, Glut1, AKT, SLC2A1, immune activation

## Abstract

Untreated HIV infection is associated with progressive CD4+ T cell depletion, which is generally recovered with combination antiretroviral therapy (cART). However, a significant proportion of cART-treated individuals have poor CD4+ T cell reconstitution. We investigated associations between HIV disease progression and CD4+ T cell glucose transporter-1 (Glut1) expression. We also investigated the association between these variables and specific single nucleotide polymorphisms (SNPs) within the Glut1 regulatory gene AKT (rs1130214, rs2494732, rs1130233, and rs3730358) and in the Glut1-expressing gene SLC2A1 (rs1385129 and rs841853) and antisense RNA 1 region SLC2A1-AS1 (rs710218). High CD4+Glut1+ T cell percentage is associated with rapid CD4+ T cell decline in HIV-positive treatment-naïve individuals and poor T cell recovery in HIV-positive individuals on cART. Evidence suggests that poor CD4+ T cell recovery in treated HIV-positive individuals is linked to the homozygous genotype (GG) associated with SLC2A1 SNP rs1385129 when compared to those with a recessive allele (GA/AA) (odds ratio = 4.67; *P* = 0.04). Furthermore, poor response to therapy is less likely among Australian participants when compared against American participants (odds ratio: 0.12; *P* = 0.01) despite there being no difference in prevalence of a specific genotype for any of the SNPs analyzed between nationalities. Finally, CD4+Glut1+ T cell percentage is elevated among those with a homozygous dominant genotype for SNPs rs1385129 (GG) and rs710218 (AA) when compared to those with a recessive allele (GA/AA and AT/TT respectively) (*P* < 0.04). The heterozygous genotype associated with AKT SNP 1130214 (GT) had a higher CD4+Glut1+ T cell percentage when compared to the dominant homozygous genotype (GG) (*P* = 0.0068). The frequency of circulating CD4+Glut1+ T cells and the rs1385129 SLC2A1 SNP may predict the rate of HIV disease progression and CD4+ T cell recovery in untreated and treated infection, respectively.

## Introduction

Metabolic dysfunction of immune cells is a hallmark of HIV infection, with implications in pathogenesis and disease progression (1–6). HIV disease progression varies considerably between individuals, with no approved methods to determine the rate of progression to AIDS or to predict favorable immunological recovery on treatment (7–12). Low CD4+ T cell count is associated with hyperactive glucose metabolism and dysregulation of glucose transporter 1 (Glut1), the major transporter of glucose on immune cells essential for CD4+ T cell activation and effector function (5, 6, 13–15). The genetic variation in immune-related genes and observed links to HIV disease progression has previously been investigated (16–22). However, the link between genetic variations in genes that regulate glucose metabolism and HIV disease progression has never been established.

Recent advances in immunometabolism support the concept that fundamental processes of metabolism in T cells are closely linked to their survival and functions (23). Indeed, susceptibility to HIV and disease progression has been associated with human leukocyte antigens HLA-B*27 and HLA-B*57, which influence the rate of CD4+ T cell loss and viral load (24–28). These clinical outcomes are dependent on the genetic diversity of specific identified key amino acid positions in the peptide-binding groove of HLA-B (29, 30). However, recent evidence has shown that viral controllers with maintained low levels of HIV replication do not always have these protective HLA allele configurations (31). This suggests new directions to expand our understanding of the mechanism of immunological dysfunctions in treated and untreated HIV infection may offer opportunities to develop novel treatments to improve immune recovery.

Immunometabolic dysfunctions related to HIV infection is associated with over expression of the glucose transporter Glut1, leading to increased influx of glucose into CD4+ T cells, irrespective of combination antiretroviral therapy (cART) status (1). Glut1 is regulated by the phosphoinositide 3-kinase (PI3K)/protein kinase B (AKT)/mammalian target of rapamycin (mTOR) pathway associated with T cell growth, proliferation and apoptosis, and more recently, HIV latency and replication (13, 32–34). As a key regulator of T cell growth and metabolic processes, abnormal PI3K/AKT/mTOR signaling is a characteristic feature of leukemia (35, 36), a disease associated with increased Glut1 expression and glucose influx in B and T cells isolated from blood and myeloid cells from bone marrow (37–39).

A posttranslational Glut1 regulator (40), AKT has five polymorphisms (rs3803300, rs1130214, rs2494732, rs1130233, and rs3730358) associated with metabolic dysfunction and the development of specific cancers, including nasopharyngeal and oral squamous cell carcinoma, non-small cell lung carcinoma, and gastric cancer (41–43). The relationship between AKT single nucleotide polymorphisms (SNPs) and systemic metabolic dysregulation has been shown by lower circulating blood glucose and lower fasting insulin levels in participants with at least one copy of the T allele found at the AKT rs1130214 site, consistent with lower insulin resistance observed in the presence of the T allele (44).

Glucose influx in breast cancer tissue associated with SNPs in SLC2A1, the gene that encodes Glut1, was attributable to the rs841853 locus in 52 German caucasians with primary breast cancer (45). Within this population, the G > T SNP is associated with upregulation of breast tumor glucose uptake compared to normal breast cells *in vivo* (45). Ng et al. (46) found expression of Glut1 Enhancer-2 SNP 1, located within putative insulin-responsive enhancer-2, was associated with diabetic nephropathy as a result of high intracellular glucose levels in response to insulin and hyperglycemia among 230 North American caucasians with type 1 diabetes.

It is now acknowledged that T cell metabolism dictates their survival, activation, differentiation, and functions. Activated T cells shift glucose metabolism toward a glycolytic phenotype reminiscent of cancer cells even in the presence of physiologically normal oxygen levels, known as the Warburg effect (1, 5). Because of this shared similarity in metabolism, SNPs regulating glucose uptake and metabolism in cancer cells may also regulate glucose metabolism in T cells. By analyzing SNPs associated with the AKT gene (rs3803300, rs1130214, rs2494732, rs1130233, and rs3730358) as well as in the Glut1 gene SLC2A1 (rs1385129 and rs841853) and antisense RNA 1 region SLC2A1-AS1 (rs710218), this study investigated the association between genes that regulate glucose metabolism and HIV disease outcome in treated and untreated HIV-positive people. This study determined whether genetic variants in metabolic genes are associated with HIV disease outcomes.

## Materials and Methods

### Study Participants

The study population included 29 HIV-positive treatment-naïve individuals, 39 HIV-positive individuals on cART (HIV+/cART), and 32 HIV seronegative controls (HIV-negative). Participating individuals were recruited from the community and the Infectious Diseases Unit at The Alfred Hospital (A state referral service for HIV care) in Melbourne, VIC, Australia. Viable peripheral blood mononuclear cells (PBMCs) were also obtained from the Clinical Research Core (CRC) Repository at the University of Washington, Seattle, WA, USA. This study was carried out in accordance with the recommendations of ethics committees at the participating institutions, with written informed consent from all subjects. All subjects gave written informed consent in accordance with the Declaration of Helsinki. The protocol was approved by the Alfred institutional board. Blood samples were collected in citrate anticoagulant tubes and processed within 1 h of venepuncture to isolate and cryopreserve PBMCs. All participants with self-reported co-infection with hepatitis C virus, active malignancy, vaccination, physical trauma, or surgery within 3 weeks prior to participation were excluded from this study.

### Peripheral Blood Mononuclear Cell Preparation

Peripheral blood mononuclear cells were isolated using density gradient centrifugation (Lymphoprep, Axis Shield, Dundee, Scotland) (47), before being cryopreserved in 10% dimethyl-sulfoxide (DMSO; Sigma-Aldrich, St. Louis, MO, USA) and 90% autologous plasma. Cryopreserved PBMCs (>90% viability) were thawed in supplemented RPMI-1640 medium [10% human serum, penicillin/streptomycin (Invitrogen), 2 mmol/L l-glutamine (Invitrogen, Carlsbad, CA, USA)], before being stained on ice for 30 min as previously described (1).

### Single Nucleotide Polymorphism Analysis

Peripheral blood mononuclear cell DNA was extracted and subjected to sequencing for SNP analysis by the Australian Genome Research Facility (QLD, Australia) using the iPLEX™ Assay (48).

### Categorization of Favorable and Non-Favorable Genotypes in HIV-Positive Individuals

Favorable or normal disease progressors not on cART are defined by having CD4+ T cell counts within the range of 200–1,500 cells/μL within the first 3 years after initial diagnosis and are maintained above 200 cells/μL within 3–7 years after initial diagnosis, or the loss of less than 80 cells/μL per year. Slow and long term non-progressors were also classified as favorable disease progressors and defined as having a CD4+ T cell count of >500 cells/μL for up to 7–10 and >10 years, respectively. Non-favorable disease progressors are defined as having CD4+ T cell counts that fell below 200 cells/μL within the first 3 years of diagnosis or experiencing a loss of >80 cells/μL per year. These criteria were adapted from previously described work (49–55).

Due to our modest sample size, we assigned very stringent published criteria for our subject groups. Thus, favorable HIV+/cART responders are defined as participants who sustained CD4+ T cell counts >500 cells/μL after at least 3 years of cART. Non-responders are defined by having a CD4+ T cell count of <500 cells/μL despite at least 3 years on cART. These criteria are as previously described (9–12).

### Flow Cytometric Analysis

Peripheral blood mononuclear cells were stained with fluorochromatic monoclonal antibodies CD3 (PE), CD4 (PerCP), and CD8 (APC) provided by BD Bioscience, and CD4 (PE Texas Red), CD57 (FITC), CD28 (PerCP Cy5.5), and PD1 (PE) from Invitrogen, ThermoFischer Scientific, before being washed and resuspended in 300 µL of 1× PBS prior to analysis with flow cytometry (BD Biosciences, San Jose, CA, USA). The Glut1 antibodies [FAB1418A and MAB1418 clones (R&D Systems, Minneapolis, MN, USA)] conjugated with APC and FITC were used to analyze Glut1 on CD4+ and CD8+ T cells using the staining procedure previously described (1). PBMCs were acquired on a FACS Calibur flow cytometer (BD Biosciences) and analyzed using FlowJo software, version 8.8 (Tree Star Inc., Ashland, OR, USA).

### Statistical Analysis

Statistical analyses were performed using STATA (Version 13.1; StataCorp, College Station, TX, USA) and SPSS (Version 23; IBM statistical software, Armock, New York, NY, USA), while GraphPad Prism (Version 6.0; GraphPad Software, La Jolla, CA, USA) was used to construct figures. A “normal range” for CD4+Glut1+ T cell percentage was established using the 5th–95th centile of results obtained on HIV-control samples.

Mann–Whitney testing was used to compare unpaired, non-parametric continuous data between patient groups, which are described using interquartile ranges (IQR) (25th–75th centiles). Comparison of groups with high and low CD4+Glut1+ T cell percentage, age, and country of origin were performed using the Fishers Exact Test between genotypes. People who were homozygous for SLC2A1 SNP rs841853 and SLC2A1-AS1 SNP rs710218 were merged into four groups of genotypic combinations (TT and AA, TT and TT, GG and AA, GG and TT) and their data compared using Mann–Whitney testing using IQR (25th–75th centiles), with homozygote dominant and recessive genotypes grouped accordingly for HIV-positive treatment naive and HIV+/cART individuals, excluding those with heterozygous genotypes. Spearman correlation coefficients were used to assess associations between non-parametric, continuous variables. *P* values <0.05 were considered significant. Univariable logistic regression analyses were performed to examine associations between response to therapy and SNPs of interest. This was followed by multivariate logistic regression modeling where we included other important HIV-related variables that could have influenced disease progression [age, body mass index (BMI), sex, CD4+ T cell count, CD4%, CD4/CD8 ratio, time on cART, viral load, or percentage of CD4+Glut1+ T cells] using a step wise removal procedure to obtain the model of best fit.

## Results

### Participant Characteristics

Participants recruited included 32 HIV-negative controls, 29 HIV-positive treatment-naïve, and 39 HIV+/cART individuals (Table 1). Controls were similar in age to both treatment-naïve HIV-positive and HIV+/cART groups (Table 1). As expected, treatment-naïve individuals had lower CD4+ T cell counts and lower CD4/CD8 ratios than those on cART, and higher HIV viral loads (Table 1). To establish a typical range, CD4+Glut1+ T cell percentage was measured in HIV-negative controls. Based on these values, a range of 0.6% (fifth centiles) to 9.4% (95th centiles), with a median CD4+Glut1+ T cell percentage value of 4.6%, was established as a standard range for CD4+Glut1+ T cell percentages to create a qualitative data set in HIV-positive individuals (Table 1).

**Table 1:** Clinical characteristics of study population.

Variables	^a^HIV-negative	^b^HIV-positive treatment-naive	^c^HIV+/cART	Mann–Whitney test
*N*	32	29	39	
Age (median)	37 (*N* = 24) [interquartile ranges (IQR) = 25.3]	44 (*N* = 22) (IQR = 13.5)	49 (*N* = 31) (IQR = 16.0)	0.40^a,b^
				0.060^a,c^
				0.087^b,c^
Country of origin (Australia/Mexico/USA)	20/12/0	8/0/21	21/0/18	
Gender (male/female)	32/0	29/0	39/0	
Race (Black/White/Latino/Asian)	0/19/12/1	6/22/1/0	2/18/1/1	
BMI	23.6 (*N* = 23) (IQR = 6.5)	26.5 (*N* = 25) (IQR = 10.2)	24.2 (*N* = 25) (IQR = 9.3)	0.55^a,b^
				0.96^a,c^
				0.56^b,c^
CD4+ T cell count (cells/μL)	–	347 (*N* = 26) (IQR = 338.3)	563 (*N* = 32) (IQR = 402.8)	**0.021^b,c^**
Viral load (U/μL)	–	107,949.0 (*N* = 25) (IQR = 186,700)	50.5 (*N* = 19) (IQR = 1,416.3)	**<0.0001^b,c^**
CD4+ T cell percentage		18.5% (*N* = 28) (IQR = 20.9)	26.3% (*N* = 37) (IQR = 36.0)	0.066^b,c^
CD4/CD8 ratio		0.21 (*N* = 27) (IQR = 0.34)	0.41 (*N* = 37) (IQR = 0.88)	**0.037^b,c^**
CD4+Glut1+ T cell percentage (<9.4/>9.4)		12/16	14/25	

*cART, combination antiretroviral therapy; USA, United States of America; BMI, body mass index*.

*Bold font indicates significant values, proven by a P value of < 0.05*.

### The *SLC2A1* SNP rs1385129 Is Associated With Immunological Response on cART but Not With Disease Progression in Treatment Naive Subjects

Using univariable logistic regression analysis among the HIV-positive treatment-naive population, unfavorable disease progression was associated with lower CD4+ T cell percentage (OR: 0.82; CI: 0.71–0.95; *P* = 0.007) and CD4/CD8 ratio (OR: <0.0001; CI: <0.0001–0.06; *P* = 0.01) (Table 2). Unfavorable disease progression was also associated with higher CD4+Glut1+ T cell percentage (OR: 1.08; CI: 1.01–1.15; *P* = 0.02) (Table 2). No SNPs were associated with disease progression among the HIV-positive treatment-naive population (*P* > 0.05).

**Table 2:** Univariate associations with disease progression among HIV-positive treatment-naïve individuals and response to therapy for HIV+/cART individuals.

Variables	HIV-positive treatment-naïve univariate analysis				HIV+/cART univariate analysis			
	Genotype (*N*)	OR	95% CI	*P*-value	Genotype (*N*)	OR	95% CI	*P*-value
Sex	–	0.67	0.04–11.94	0.78	–	0.68	0.04–11.95	0.80
Age	–	1.11	0.98–1.26	0.10	–	0.99	0.93–1.06	0.85
BMI	–	1.05	0.95–1.16	0.34	–	0.92	0.80–1.06	0.27
Race (Australian/USA)	–	0.16	0.02–1.51	0.11	–	**0.12**	**0.02–0.60**	**0.01**
CD4+ T cell count (cells/μL)	–	0.97	0.94–1.01	0.10	–	0.97	0.93–1.00	0.08
CD4+ T cell percentage	–	**0.82**	**0.71–0.95**	**0.007**	–	**0.92**	**0.87–0.98**	**0.004**
CD4/CD8 ratio	–	**<0.0001**	**<0.0001–0.06**	**0.01**	–	**0.02**	**0.001–0.36**	**0.008**
CD4+Glut1+ T cell percentage	–	**1.08**	**1.01–1.15**	**0.02**	–	**1.14**	**1.02–1.27**	**0.03**
Years on cART	–	–	–	–	–	0.95	0.84–1.07	0.40

SLC2A1 SNPs								
rs1385129	GG = 13	1.89	0.41–8.61	0.41	**GG = 22**	**4.67**	**1.04–20.94**	**0.04**
	GA/AA = 15				**GA/AA = 17**			

rs841853	TT = 13	0.34	0.07–1.77	0.20	TT = 18	1.18	0.32–4.42	0.80
	TG/GG = 14				TG/GG = 20			

**SLC2A1-AS1 SNP**								
rs710218	AA = 9	0.75	0.14–3.90	0.73	AA = 20	2.29	0.60–8.83	0.23
	AT/TT = 19				AT/TT = 19			

**AKT SNPs**								
rs3730358	CC = 18	2.13	0.42–10.78	0.36	CC = 27	0.7	0.17–2.84	0.62
	CT/TT = 10				CT/TT = 12			

rs3803300	GG = 22	3.08	0.30–31.98	0.35	GG = 33	0.48	0.06–3.86	0.49
	GA/AA = 5				GA/AA = 4			

rs1130214	GG = 8	0.35	0.06–2.12	0.25	GG = 14	0.35	0.08–1.56	0.17
	GT/TT = 20				GT/TT = 25			

rs1130233	GG = 14	1.50	0.33–6.77	0.60	GG = 21	0.79	0.21–2.92	0.72
	GA/AA = 14				GA/AA = 18			

rs2494732	CC = 9	0.35	0.06–2.12	0.25	CC = 5	1.22	0.18–8.36	0.84
	CT/TT = 19				CT/TT = 34			

*cART, combination antiretroviral therapy; BMI, body mass index; USA, United States of America*.

*Bold font indicates significant values, proven by a P value of < 0.05*.

Univariable logistic regression was next used to assess associations with immunological response to cART among the HIV+/cART population. Poor response to therapy was less likely among Australian participants when compared against American participants (OR: 0.12; CI: 0.02–0.60; *P* = 0.01) (Table 2). As expected, poor response to therapy was inversely associated with CD4+ T cell percentage (OR: 0.92; CI: 0.87–0.98; *P* = 0.004) and CD4/CD8 ratio (OR: 0.02; CI: 0.001–0.36; *P* = 0.008) (Table 2). Furthermore, a poor response to therapy was associated with increased CD4+Glut1+ T cell percentage (OR: 1.14; CI: 1.02–1.27; *P* = 0.03) (Table 2). When analyzing SNPs, people with the dominant GG genotype associated with SLC2A1 SNP rs1385129 had a 4.67-fold higher risk of poor response to therapy (CI: 1.04–20.94; *P* = 0.04) when compared to those with a GA or AA genotype (Table 2).

To better understand the inverse relationship between CD4+ T cell counts and Glut1 expression on these cells, we evaluated Glut1 expression on exhausted CD4+ T cells marked by PD1 expression (56) and on CD4+ T cells with a senescence phenotype (CD57+CD28−) (57). We show that Glut1 is significantly elevated on cells with an exhausted and senescent phenotype, indicating that these CD4+ T cells are metabolically exhausted as previously hypothesized (6) (Figure S1 in Supplementary Material).

Using multivariate logistic regression modeling, it was shown that the GG genotype of SNP rs1385129 and lower total CD4+ T cell percentage, independent of each other, were dependently associated with poor response to therapy (OR: 0.08; CI: 0.01–0.7; *P* = 0.02; and OR: 0.9; CI: 0.8–1.0; *P* = 0.003, respectively) independent of age, BMI, sex, and time on cART. No other variables of interest were associated with any SNPs, progression to disease, or recovery with treatment (*P* > 0.05). Further multivariable analysis was limited due to low participant numbers.

### CD4+Glut1+ T Cell Percentage Is Highest in HIV-Positive Individuals With Non-Favorable Disease Progression and Poor Response to cART

**Figure 1:**
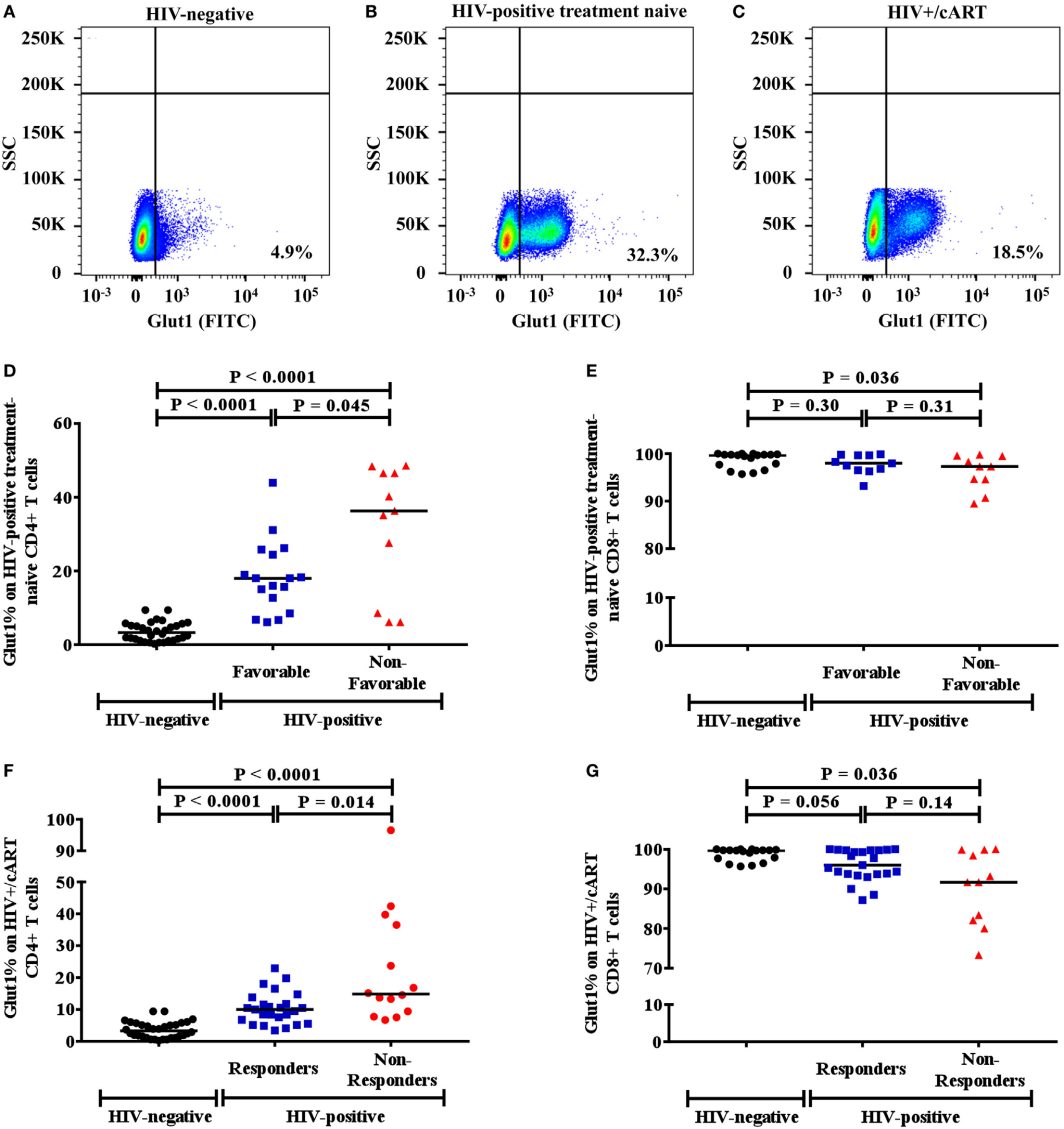
Gaiting strategy and analysis of CD4+Glut1+ T cell percentage in HIV-negative and HIV-positive populations. **(A–C)** Percentage of Glut1 expressing CD3+CD4+ T cells within the lymphocyte population were defined using side scatter (SSC) and the Glut1 fluorophore (FITC); **(D,E)** comparison between HIV-negative controls and HIV-positive treatment-naïve groups with favorable and non-favorable disease progression; **(F,G)** comparison between HIV-negative controls and HIV+/cART responder and non-responder groups. Horizontal black lines refer to median CD4+Glut1+ or CD8+Glut1+ T cell percentages of each group. cART, combination antiretroviral therapy.

Since high Glut1 expression on CD4+ T cells has previously been implicated in low CD4+ T cell counts in both HIV-positive treatment-naive and cART-treated patients (1), we first determined whether an association existed between the frequency of circulating CD4+ T cells expressing Glut1 and both unfavorable disease progression and poor CD4+ T cell recovery in our cohort. The percentage of CD4+ T cells expressing Glut1, a surrogate marker of T cell activation and glycolytic metabolism, was compared between controls and HIV-positive treatment-naïve individuals. A conventional gating strategy was used to evaluate CD4+Glut1+ T cell percentage (Figures 1A–C). The percentages of CD4+Glut1+ T cells were higher for both favorable (median: 18.0%; IQR = 14.5) and non-favorable (median: 36.3%; IQR = 37.9) disease progression in untreated individuals when compared to HIV-negative controls (median: 4.6%; IQR = 4.9) (*P* < 0.0001) (Figure 1D). Furthermore, CD4+Glut1+ T cell percentage was higher in HIV-positive treatment-naïve individuals with non-favorable progression when compared to those with favorable progression (*P* = 0.045) (Figure 1D). HIV-negative controls also had significantly higher percentages of CD8+Glut1+ T cells when compared to those with non-favorable progression (*P* = 0.036) but were similar when compared to favorable progressors (median: 98.0; IQR = 3.2) (*P* = 0.30) (Figure 1E). The percentage of CD8+Glut1+ T cells was not statistically different between HIV-positive groups (*P* = 0.31) (Figure 1E).

The percentage of CD4+Glut1+ T cells was also higher in cART-treated immunological responder (median: 10.0%; IQR = 6.6) and non-responder populations (median: 14.8%; IQR = 28.3) when compared to controls (median: 4.6%; IQR = 4.9) (*P* < 0.0001) (Figure 1F). Furthermore, CD4+Glut1+ T cell percentages were higher in immunological non-responders than in immunological responders (*P* = 0.014) (Figure 1F). HIV-negative controls had similar percentages of CD8+Glut1+ T cells (median: 99.7; IQR = 2.6) when compared to immunological responders (median: 96.0; IQR = 6.1) (*P* = 0.056) (Figure 1G). However, immunological non-responders had lower CD8+Glut1+ T cell percentages (median: 91.7; IQR = 17.8) when compared to controls (*P* = 0.036) (Figure 1G).

Mann–Whitney testing assessed if participant characteristics varied between favorable and non-favorable groups in HIV-positive individuals (Table S1 in Supplementary Material). CD4+ T cell counts, CD4+ T cell percentages, and CD4/CD8 ratios were highest in those with favorable disease progression and recovery (Table S1 in Supplementary Material). Viral loads were also highest among non-favorable HIV-positive treatment-naïve individuals (Table S1 in Supplementary Material).

### CD4+Glut1+ T Cell Percentage Correlates Inversely With CD4+ T Cell Counts and Percentage in Those With HIV Infection

**Figure 2:**
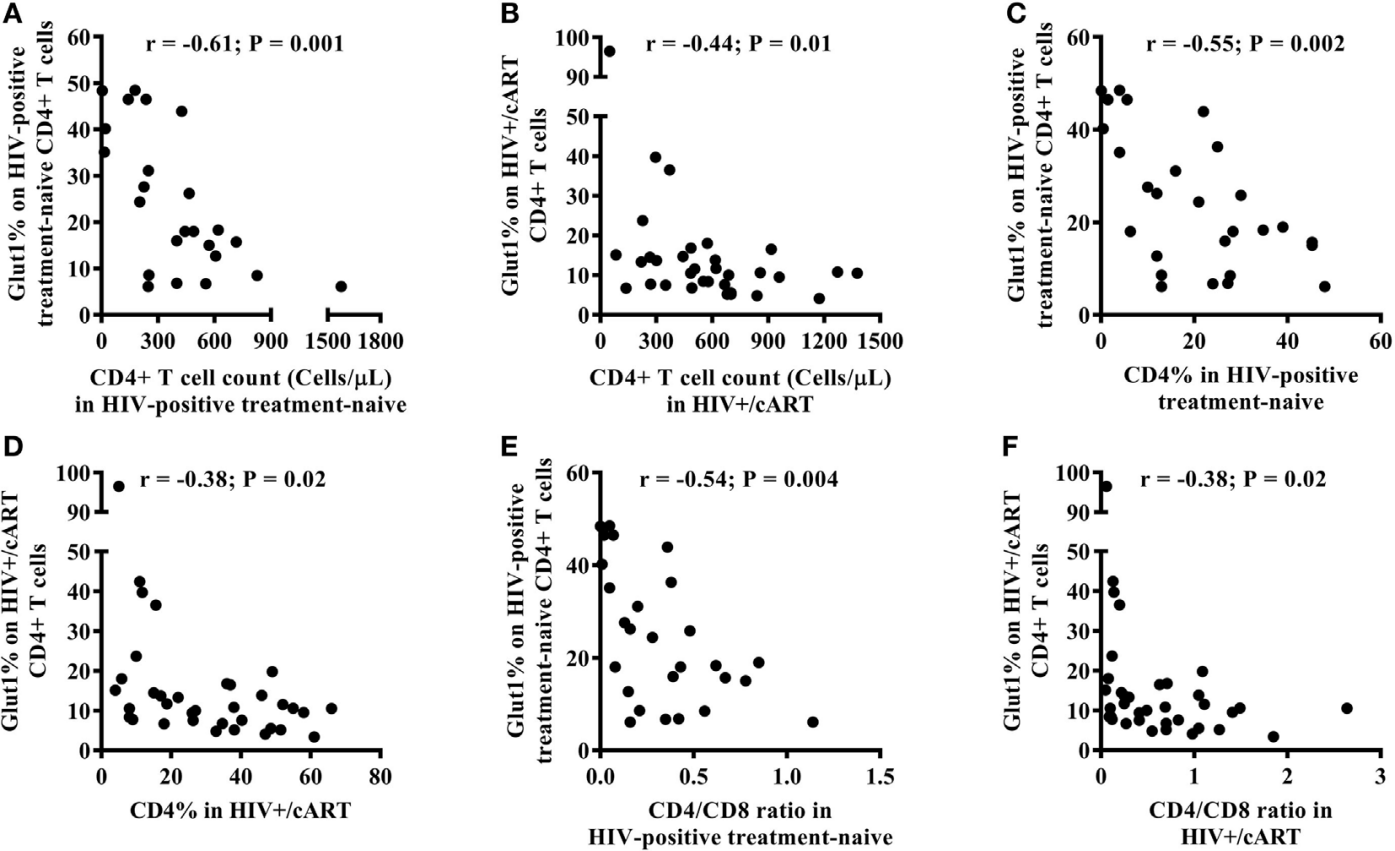
CD4+Glut1+ T cell percentage inversely correlates with CD4+ T cell counts **(A,B)**, CD4+ T cell percentage **(C,D)**, and CD4/CD8 ratio **(E,F)** in HIV-positive individuals confirming previous results (1). cART, combination antiretroviral therapy.

Among HIV-positive treatment-naïve and HIV+/cART populations, there was an inverse relationship between the frequency of circulating CD4+Glut1+ T cells and that of total CD4+ T cell counts (Figures 2A,B). This relationship also existed between the percentage of CD4+Glut1+ T cells and total CD4+ T cell percentage confirming previous findings (Figures 2C,D) (1). CD4+Glut1+ T cell percentage was also inversely correlated with the CD4/CD8 ratio in HIV-positive treatment-naïve and HIV+/cART individuals (Figures 2E,F). We found no association between age, BMI, viral load, or duration of cART use and the percentage of CD4+Glut1+ T cells.

### CD4+Glut1+ T Cell Percentage Is Different Between Genotypes Associated With *SLC2A1* and *AKT* SNPs in HIV+/cART Individuals

**Figure 3:**
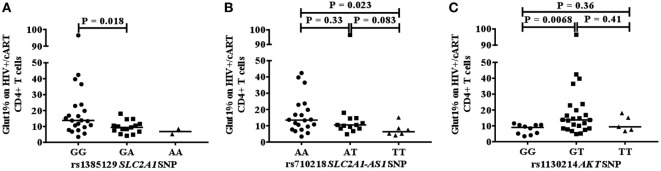
Association between CD4+Glut1+ T cell percentage and genetic polymorphisms associated with the SLC2A1 SNPs rs1385129 **(A)** SLC2A1-AS1 SNP rs710218 **(B)**, AKT SNP rs1130214 **(C)**. cART, combination antiretroviral therapy; SNP, single nucleotide polymorphisms.

We next investigated whether a relationship exists between the frequency of circulating CD4+Glut1+ T cells and SNPs within genes that encode the Glut1 protein (SLC2A1) and AKT. The percentage of CD4+Glut1+ T cells was compared between allele populations in HIV+/cART individuals. The dominant homozygote allele (GG) for the rs1385129 SLC2A1 SNP was associated with higher CD4+Glut1+ T cell percentage in HIV+/cART individuals at 13.8% (IQR = 14.0) when compared to the heterozygous (GA) allele percentage of 9.4% (IQR = 4.8; *P* = 0.018) (Figure 3A). A median value of 6.8% was obtained in individuals with the recessive homozygote allele (AA). There was no difference in percentage of CD8+Glut1+ T cells between those with the homozygous dominant genotype and those with a recessive allele (GG: median: 99.6%; IQR = 7.2 vs. GA/AA: median: 94.2%; IQR = 8.5; *P* = 0.096).

When analyzing the rs710218 SLC2A1-AS1 SNP in HIV+/cART individuals, the dominant homozygote allele (AA) was associated with higher CD4+Glut1+ T cell percentage at 13.5% (IQR = 14.0) when compared to the recessive homozygous (TT) percentage of 6.3% (IQR = 5.2; *P* = 0.023) (Figure 3B). No difference was found between the heterozygous (AT) median at 10.5% (IQR = 6.3) and the dominant (*P* = 0.33) and recessive homozygous medians (*P* = 0.083) (Figure 3B). The median percentage of CD4+Glut1+ T cells among homozygous dominant individuals was also higher when compared to those with a recessive allele (AT or TT) suggesting a higher percentage of CD4+ T cell metabolic activation in HIV+/cART individuals with the rs710218 GG genotype (*P* = 0.04). Percentage of CD8+Glut1+ T cells was higher among those with a homozygous dominant genotype (GG: median: 99.8%) when compared to those with a recessive allele (GA/AA: median: 93.9%; *P* = 0.047).

The heterozygotic genotype (GT) for AKT SNP rs1130214 was associated with higher CD4+Glut1+ T cell percentage of 13.8% (IQR = 13.8) when compared with the dominant homozygous genotype (GG) (median: 9.0%; IQR = 5.6) (*P* = 0.0068) (Figure 3C). There was no difference between the recessive homozygous population (TT) value of 9.4% (IQR = 9.4) and the values of the other genotypes (Figure 3C). Therefore, the GT genotype of the rs1130214 AKT SNP is associated with persistently high CD4+ T cell glycolytic activity in HIV-positive cART-treated individuals.

### CD4+Glut1+ T Cell Percentage Is Highest in HIV+/cART Individuals With Dominant Homozygous Genotypes for Both rs841853 *SLC2A1* and rs710218 *SLC2A1-AS1* SNPs

Based on the 1,000 genomes data, which describes the linkage disequilibrium between SLC2A1 SNP rs841853 and SLC2A1-AS1 SNP rs710218 to be strongly linked across most individuals (58), subjects were merged into four groups. Homozygote dominant and recessive genotypes were grouped accordingly for both HIV-positive treatment-naïve and HIV+/cART groups, excluding those with heterozygous genotypes for both SNP positions. HIV+/cART individuals with a dominant homozygous genotype at positions rs841853 (TT) and rs710218 (AA) had a higher CD4+Glut1+ T cell percentage median of 11.6% (*N* = 26; IQR = 9.4) when compared to the 8.3% (*N* = 11; IQR = 3.8) found among those with a dominant homozygous genotype at position rs841853 (TT) and a recessive homozygous genotype at rs710218 (TT) (*P* = 0.02). Despite being associated with CD4+Glut1+ T cell percentage, HIV+/cART individuals with dominant homozygous genotypes for rs841853 and rs710218 were not associated with disease progression.

### The *SLC2A1* SNP 1385129 Is Associated With High CD4+Glut1+ T Cell Percentage and the Non-Responder Phenotype in HIV+/cART Individuals With a Dominant Homozygous Genotype

A value of 9.4% (The 95th centile observed in normal controls) was used as the “cut off” between normal and elevated results in the analysis of HIV-positive treatment-naïve and HIV+/cART participants and used to assess if high and normal CD4+Glut1+ T cell percentages are associated with allele sets attributed to AKT and SLC2A1 SNPs. Among all SLC2A1 SNPs analyzed, only rs1385129 was found to be associated with CD4+Glut1+ T cell percentage in HIV-positive individuals on cART. Those with the GG allele were more likely to have high CD4+Glut1+ T cell percentage than those with the GA/AA allele sets (*P* = 0.038) (Table 3).

**Table 3:** Fisher’s exact testing of rs1385129 SLC2A1 single nucleotide polymorphism allele distribution against high and normal HIV+/cART CD4+Glut1+ T cell percentage groups, and responder and non-responder groups.

rs1385129	GG	GA/AA	Total
CD4+Glut1+ T cell percentage <9.4	4	9	13
CD4+Glut1+ T cell percentage >9.4	16	7	23
Total	20	16	36
Fisher’s exact test (*P-*value) = 0.038			

HIV + /cART responder	11	11	22
HIV + /cART non-responder	14	3	17
Total	25	14	39
Fisher’s exact test (*P-*value) = 0.049			

*cART, combination antiretroviral therapy*.

The association between SLC2A1 rs1385129 and favorable treatment response phenotypes in HIV+/cART individuals was confirmed using Fisher’s exact testing. Immunological non-response was more commonly seen in those with the GG genotype than in those with GA and AA allele sets (*P* = 0.049) (Table 3). We found no associations between percentage of CD4+Glut1+ T cells and clinical variables including age, BMI, time on cART, CD4+ T cell percentage, and CD4/CD8 ratio in HIV+/cART individuals.

SLC2A1 rs1385129 was analyzed among participants from Australia and America in HIV+/cART individuals using Fisher’s exact testing. Specific rs1385129 genotypes were not more common in those from either country (*P* = 0.21) (Table 4). However, unfavorable treatment response was associated with participants from the USA independently of CD4+Glut1+ T cell percentages (*P* = 0.024) (Table 4). No significant results were found when analyzing other SNP genotypes among either HIV-positive group (*P* > 0.05).

**Table 4:** Fisher’s exact testing of country of origin against genotypes associated with the rs1385129 SLC2A1 single nucleotide polymorphism and responder and non-responder HIV+/cART groups.

	Australia	USA	Total
GG	14	8	22
GA/AA	7	10	17
Total	21	18	39
Fisher’s exact test (*P* value) = 0.21			

HIV+/cART responder	17	8	25
HIV+/cART non-responder	4	10	14
Total	21	18	39
Fisher’s exact test (*P* value) = 0.024			

*cART, combination antiretroviral therapy; USA, United States of America*.

### Elevated CD4+Glut1+ T cell Percentage Is Associated With Non-Favorable Disease Progression Among Those With *AKT* And *SLC2A1* SNP Genotypes That Maintain Hardy–Weinberg Equilibrium

We assessed CD4+Glut1+ T cell percentage between favorable and non-favorable HIV-positive individuals with specific genotypes, employing Hardy–Weinberg equilibrium to ensure that allele frequencies remain constant. The GG genotype of SLC2A1 SNP rs1385129 was associated with a higher CD4+Glut1+ T cell percentage among non-favorable progressors (median: 43.4%; IQR = 11.0) compared with favorable progressors among HIV-positive treatment-naïve individuals (median: 16.0%; IQR = 11.7; *P* = 0.0082) (Hardy–Weinberg equilibrium = 0.47; *P* = 0.49) (Table S2 in Supplementary Material; Figure S2A in Supplementary Material). The dominant homozygous rs710218 SNP population (AA) had a higher CD4+Glut1+ T cell percentage (median: 46.5%; IQR = 1.9) in non-favorable progressors compared with favorable progressors (median: 15.8%; IQR = 18.2; *P* = 0.024) (Hardy–Weinberg equilibrium = 0.85; *P* = 0.36) (Table S2 in Supplementary Material; Figure S2B in Supplementary Material). Therefore, these results suggest that higher CD4+Glut1+ T cell percentage is associated with non-favorable disease progression and poor response to therapy in HIV-positive people.

The GG genotype of AKT SNP rs1130214 was associated with a higher CD4+Glut1+ T cell percentage of 39.7% among non-responders (IQR = 28.7) when compared with 9.5% for responders among HIV+/cART individuals (IQR = 5.5; *P* = 0.011) (Hardy–Weinberg equilibrium = 0.27; *P* = 0.60) (Table S2 in Supplementary Material).

## Discussion

This study investigated the associations between the frequency of circulating CD4+Glut1+ T cells, HIV disease progression in untreated and cART-treated individuals, and SNPs associated with Glut1 expression and regulation. The SNPs we studied include five of the AKT gene, two of the SLC2A1 gene, and one belonging to SLC2A1-AS1. Our analysis shows that CD4+Glut1+ T cell percentage was significantly elevated in HIV-positive treatment-naïve individuals who experienced rapid CD4+ T cell loss and in those who experienced poor CD4+ T cell recovery on cART. Among HIV+/cART individuals, high CD4+Glut1+ T cell percentages were observed in those with the dominant homozygous genotype associated with SLC2A1 SNP rs1385129, SLC2A1-AS1 SNP rs710218 and those who were heterozygous (GT) for AKT rs1130214. Although genotypes associated with rs710218 and rs1130214 were associated with Glut1 expression, they did not achieve a significant relationship with clinical outcomes, perhaps due to our modest sample size.

Elevated CD4+Glut1+ T cell percentage was previously reported among HIV-positive people when compared to HIV-negative controls regardless of treatment status (1). We show that CD4+Glut1+ T cell percentage is associated with rapid CD4+ T cell decline during untreated HIV infection, and in patients with poor CD4+ T cell recovery despite sustained viral suppression on cART. Glycolysis and oxidative phosphorylation are the primary pathways used by T cells to fulfill their energy requirements when activated (59–61). We found that Glut1, a marker of glycolytic activation, is elevated on CD4+ T cells that exhibit an exhausted and senescent phenotype. Thus, these observations support the model that elevated glycolysis facilitates metabolic exhaustion that drives CD4+ T cell loss (5, 6). Indeed, Glut1 expression on CD4+ T cells is associated with increased metabolic activity, including upregulated glucose influx, increased ROS production, and cell stress (1, 59–62).

High T cell exhaustion measured by PD-1 surface expression, and immune activation measured by the percentage of CD4+ and CD8+ T cells co-expressing the activation markers CD38 and HLA-DR have been shown in HIV+ individuals with suboptimal CD4+ T cell recovery on ART (63). Here, we demonstrate that elevated glucose metabolic activity in CD4+ T cells is observed in exhausted and senescent CD4+ T cells from cART-treated and virologically suppressed HIV+ subjects. While the mechanistic relationship between metabolism and T cell exhaustion is under-researched, it has been shown that elevated glycolytic metabolism is a common feature in senescent fibroblasts (64).

Despite prevailing knowledge that central memory CD4+ T cells constitute the major reservoir (65), other subsets like naïve T cells have been shown to harbor considerable HIV reservoirs and indeed may be major virion sources following treatment interruption (66). Naïve T cells have historically been described as immunologically resting (67), but we have recently shown that these cells are in fact metabolically active (68). The role of Glut1 in reservoir maintenance is unexplored; however, Glut1 is shown to be a facilitator of HIV infection (15, 69), with a marginal increase in total HIV DNA in CD4+Glut1+ T cells compared to CD4+Glut1− T cells in HIV+ individuals (15). Nevertheless, it appears that Glut1 expression on the majority of CD4+ T cells may be due to inflammatory signals like IL-7, known to regulate Glut1 trafficking to the cell surface of T cells (59, 70). The hypothesis that SNPs within metabolic genes influences HIV reservoir size is untested.

Our results highlight a potential link between genetic polymorphisms within genes that control glucose metabolism in CD4+ T cells. Indeed, cancerous T cells over express Glut1 to meet their requirements for glucose and drive glycolytic metabolism (39). In the context of cancer cells, this increased glycolytic metabolism is essential for biomass production and cell growth. This metabolic state is similar in activated T cells, but in the context of HIV infection, persistent activation of glycolysis in CD4+ T cells occurs at the expense of ATP production by oxidative phosphorylation. This might partly explain why HIV-positive subjects have elevated CD4+Glut1+ T cell percentage, and why we found a strong relationship between the SLC2A1 rs1385129 GG genotype and poor CD4+ T cell recovery in cART-treated patients.

**Figure 4:**
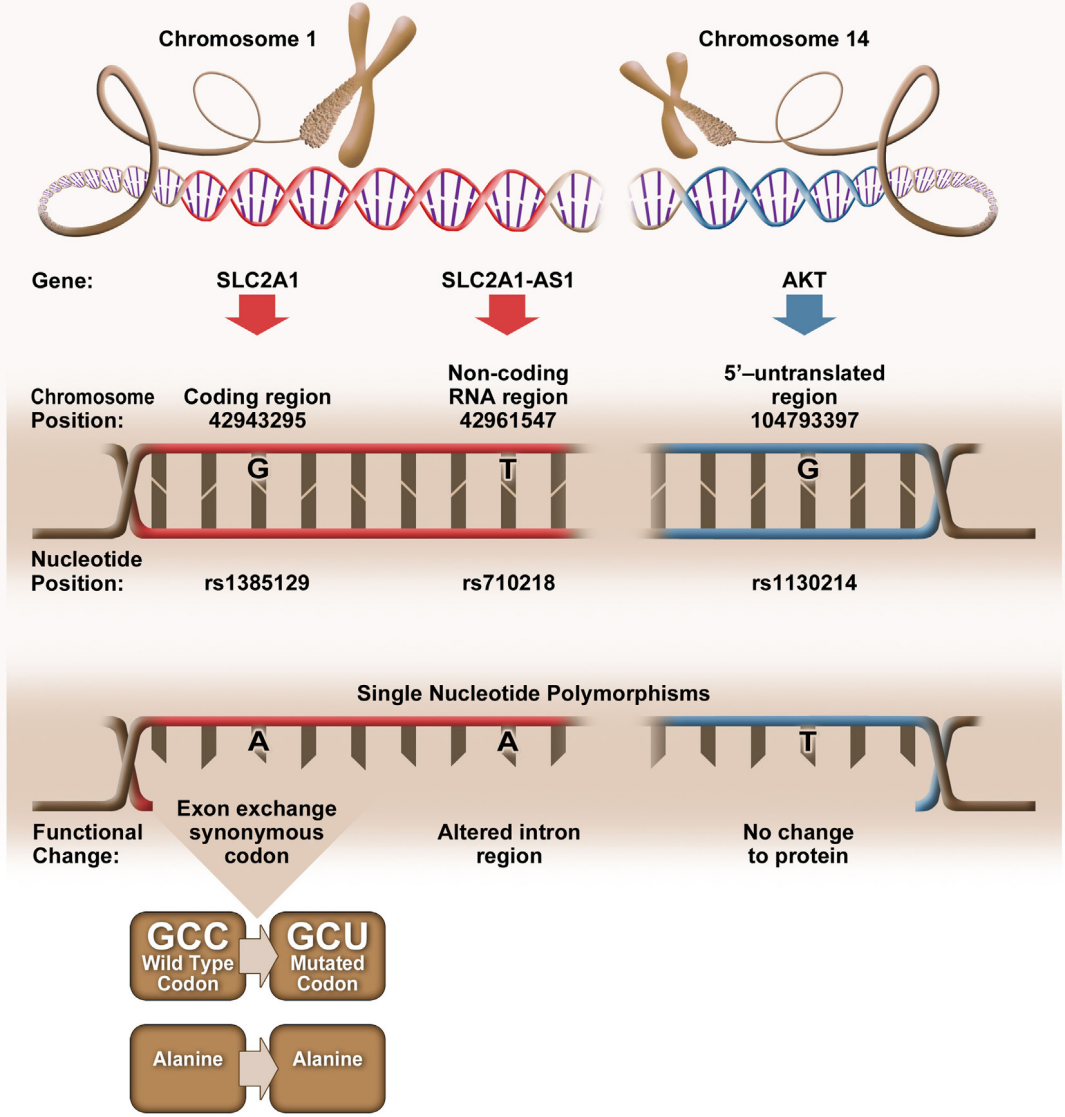
Genetic positioning and functional changes associated with SLC2A1 single nucleotide polymorphism (SNP) rs1385129 (73), SLC2A1-AS1 SNP rs710218 (74), and AKT SNP rs1130214 (75).

Bioinformatic analysis revealed that elevated CD4+Glut1+ T cell percentage was associated with SNPs (rs1385129, rs710218, rs1130214) that do not alter the amino acid sequence (Figure 4). When analyzing SLC2A1 SNP rs1385129 and SLC2A1-AS1 SNP rs710218, the dominant homozygous genotype was associated with elevated CD4+Glut1+ T cell percentage in HIV+/cART individuals. These SNPs have been studied in breast cancer cells through the measurement of fluorescently tagged glucose molecules 18F-FDG; however, there was no association between glucose uptake and these genotypes (45). However, the rs710218 SLC2A1-AS1 SNP has recently been shown to be associated with increased Glut1 expression in colorectal cancer tissue when at least one copy of the T allele was present in comparison to those with homozygous AA genotypes among individuals with colorectal cancer (71). These results do not correspond with our own, as we found non-favorable progression among the HIV-positive treatment-naive and elevated CD4+Glut1+ T cell percentage in HIV+/cART individuals to be associated with the rs710218 dominant A allele (71). Furthermore, other studies have shown that this SNP does not influence 18F-FDG uptake in breast cancer (72), suggesting that regulation of Glut1 expression by SNPs may be cell and context specific.

The rs1385129 SLC2A1 SNP is a synonymous mutation, resulting in no change in the amino acid sequence of the expressed protein. We are unable to fully explain the relationship between Glut1 expression and this SNP, since we did not evaluate SLC2A1 mRNA or total Glut1 levels in CD4+ T cells from our subjects. However, silent mutations have been shown to affect gene expression by influencing the binding of DNA regulatory factors, the structure and stability of mRNA, ribosome trafficking and its interactions with mRNA, and specific ligands that influence RNA and protein expression (76). The mechanism underlying the associations between the synonymous mutations, Glut1 expression, and the immunological outcomes in HIV+ subjects warrants further investigation.

Elevated CD4+Glut1+ T cell percentage was associated with AKT SNP 1130214, suggesting that variation within the 5’ untranslated region of the AKT gene affects Glut1 expression despite no change to the amino acid sequence (Figure 4). The Nef HIV protein binds and promotes the phosphorylation of the AKT protein in CD3+ T cells (77), with resulting hyperphosphorylation of AKT occurring with the activation of HIV-1 expression in CD4+ T cells and coinciding with the proliferation of T cells (78, 79). With HIV infection linked to upregulation of both AKT and Glut1 expression in CD4+ T cells (80), we can assume that Glut1 expression and trafficking to the cell surface is regulated upstream by AKT, a phenotype shared by cells belonging to the lymphoid cell line (81). Therefore, the frequency of circulating CD4+Glut1+ T cells, and inherited SNPs within the AKT encoding gene could be explored to identify HIV-positive untreated individuals prone to unfavorable disease progression or poor T cell recovery in cART-treated persons.

We report that non-favorable HIV disease outcomes is associated with increased frequency of circulating CD4+Glut1+ T cells linked to rapid CD4+ T cell depletion and poor recovery in untreated and treated HIV-positive persons. Among HIV+/cART populations, the homozygous dominant genotypes associated with SLC2A1 SNP rs1385129 and SLC2A1-AS1 SNP rs710218 were associated with elevated Glut1 expression, while the rs1385129 SNP GG genotype was linked with both high CD4+Glut1+ T cell percentage and non-favorable response to therapy.

Our modest sample size constitutes a limitation of this work and could be strengthened by analysis of multiple time points prior to and during cART treatment. Notwithstanding, we demonstrate that genes, which regulate glucose metabolism in T cells may influence immunological and clinical outcomes in HIV infection and support further analysis of other genes implicated in glucose metabolism in T cells such as mTOR, PI3Kinase, and HIF1. As we found that unfavorable treatment response was associated with those from the USA when compared to Australia, we conclude that differences in treatment regimens or health care between these nations warrants further investigation on their effects on immune recovery. Furthermore, the different ethnic make ups of this cohort may have influenced the distribution of SNPs analyzed, which could be explored with a larger cohort focused on specific racial backgrounds. Our results support the impact of metabolic genes in influencing immune cell biology and the course of HIV disease. Furthermore, immunometabolic SNPs could potentially be exploited to predict response to metabolic-based immunotherapy.

## Ethics Statement

This study was carried out in accordance with the recommendations of ethics committees at the participating institutions, with written informed consent from all subjects. All subjects gave written informed consent in accordance with the Declaration of Helsinki. The protocol was approved by the Alfred institutional board.

## Author Contributions

CP conceived the project, designed, conducted experiments, analysed, interpreted data and wrote the manuscript. IS-O provided samples, provided critical suggestions and reviewed the manuscript. JJRM analysed, interpreted data, wrote the manuscript and replied to reviewers comments. JJRM and TH performed bioinformatic analysis. CC analysed and interpreted data. NM, RP, BB, TH, JM and SC interpreted data, made critical intellectual suggestions, reviewed, edited and approved the manuscript, and assisted in replying to reviewers comments. JM and AL designed experiments, interpreted data and approved the manuscript.

## Conflict of Interest Statement

The authors declare that the research was conducted in the absence of any commercial or financial relationships that could be construed as a potential conflict of interest.

## Supplementary Material

Figure S1 Gaiting strategy used to analyse CD4+ T cell populations phenotypically distinct by their CD57 and CD28 surface expression **(A**,**B)**. Glut1 MFI expression in CD4+ T cells with naïve (CD57–CD28+), activated (CD57+CD28+) and senescent phenotypes (CD57+CD28–) **(C)**. Gaiting strategy used to analyse CD4+PD1– and CD4+PD1+ T cell populations **(D)**. Glut1 MFI expression in CD4+ T cells with normal (CD4+PD1–) and exhausted (CD4+PD1+) phenotypes **(E)**.

Figure S2 Genotypes and the distribution of favorable and non-favorable CD4+Glut1+ T cell percentages in HIV-positive individuals with SLC2A1 SNP rs1385129 **(A)**, and SLC2A1-AS1 SNP rs710218 **(B)**. SNP = Single nucleotide polymorphism.
