# Supplementary figures and images for: Polymorphism rs1385129 Within Glut1 Gene SLC2A1 Is Linked to Poor CD4+ T Cell Recovery in Antiretroviral-Treated HIV+ Individuals
Source: Front Immunol. 2018 May 17;9:900. doi: 10.3389/fimmu.2018.00900 (PMC5966582; doi:10.3389/fimmu.2018.00900)

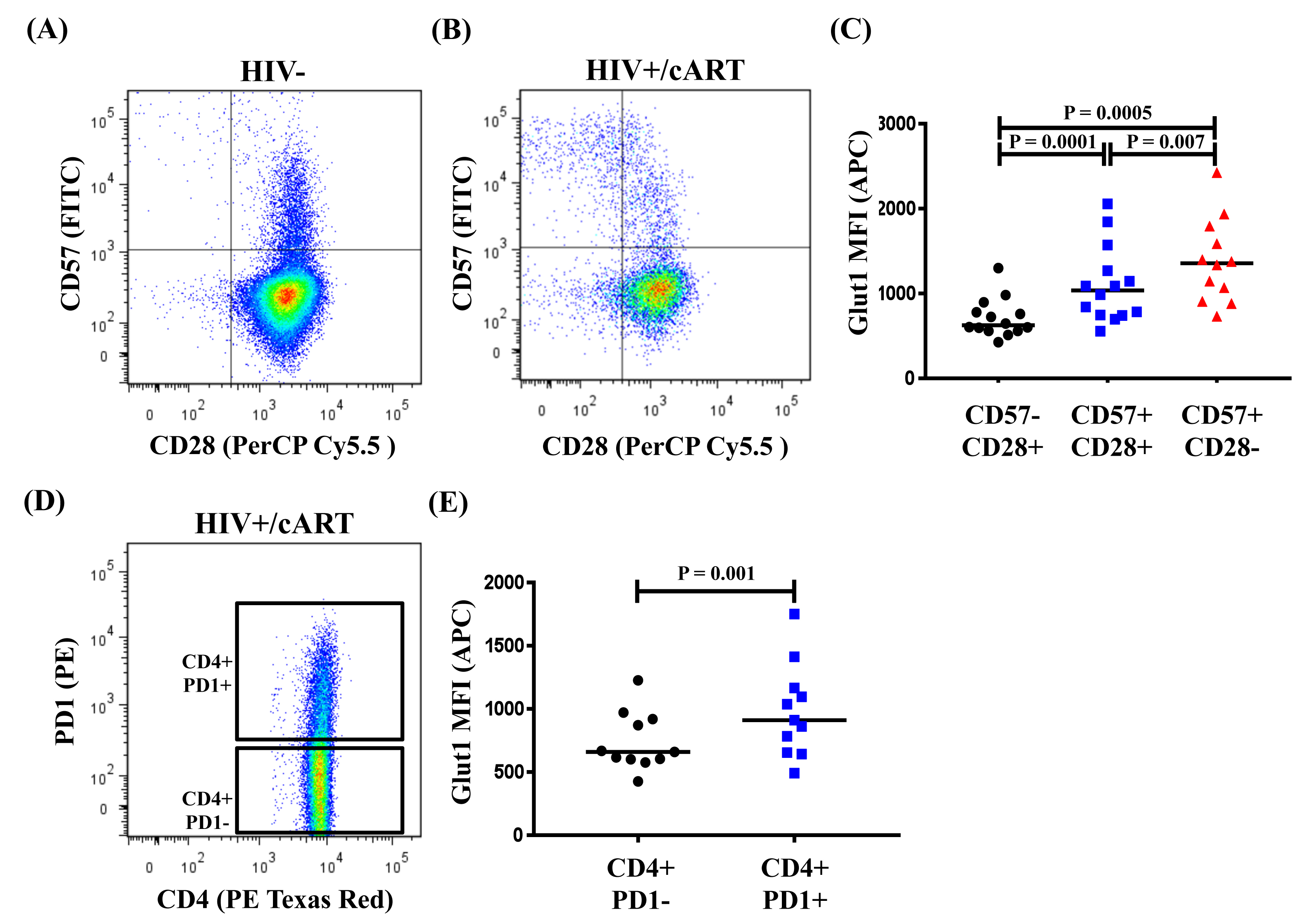

Supplement: Figure S1 — Gaiting strategy used to analyse CD4+ T cell populations phenotypically distinct by their CD57 and CD28 surface expression (A,B). Glut1 MFI expression in CD4+ T cells with naïve (CD57–CD28+), activated (CD57+CD28+) and senescent phenotypes (CD57+CD28–) (C). Gaiting strategy used to analyse CD4+PD1– and CD4+PD1+ T cell populations (D). Glut1 MFI expression in CD4+ T cells with normal (CD4+PD1–) and exhausted (CD4+PD1+) phenotypes (E). [file image_1.jpeg]

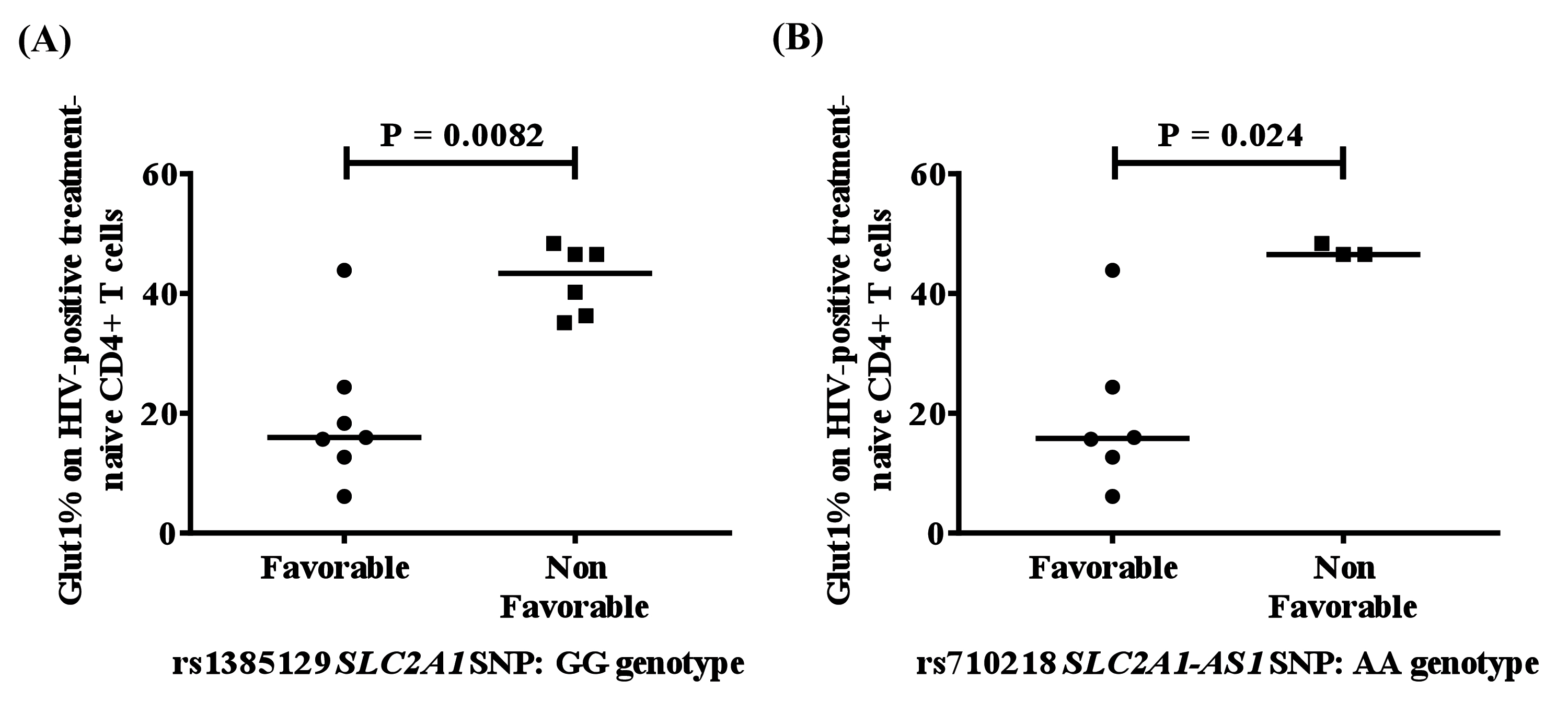

Supplement: Figure S2 — Genotypes and the distribution of favorable and non-favorable CD4+Glut1+ T cell percentages in HIV-positive individuals with SLC2A1 SNP rs1385129 (A), and SLC2A1-AS1 SNP rs710218 (B). SNP = Single nucleotide polymorphism. [file image_2.jpeg]
