# Supplementary material for: Polymorphism rs1385129 Within Glut1 Gene SLC2A1 Is Linked to Poor CD4+ T Cell Recovery in Antiretroviral-Treated HIV+ Individuals
Source: Front Immunol. 2018 May 17;9:900. doi: 10.3389/fimmu.2018.00900 (PMC5966582; doi:10.3389/fimmu.2018.00900)
Supplement: Supplementary file 3 [file data_sheet_1.docx]

**Supplementary table 1.** Analysis of variables between HIV-positive favorable and non-favorable groups.

|  | **HIV-positive treatment-naïve** | | | **HIV+/cART** | | |
| --- | --- | --- | --- | --- | --- | --- |
|  | **Favorable** | **Non-favorable** | **Mann-Whitney P value** | **Favorable** | **Non-favorable** | **Mann-Whitney P value** |
| **Age** | 42.5` | 49.0 |  | 49.0 | 46.5 |  |
|  | (N = 14) | (N = 9) | 0.098 | (N = 19) | (N = 12) | 0.73 |
|  | (IQR = 9.3) | (IQR = 13.5) |  | (IQR = 16.0) | (IQR = 16.0) |  |
| **BMI** | 25.1 | 28.4 |  | 25.0 | 24.0 |  |
|  | (N = 16) | (N = 9) | 0.17 | (N = 15) | (N = 10) | 0.39 |
|  | (IQR = 9.7) | (IQR = 13.1) |  | (IQR = 8.3) | (IQR = 9.0) |  |
| **CD4+ T cell count (Cells/µL)** | 478.0 | 202.5 |  | 677 | 268 |  |
|  | (N = 16) | (N = 10) | **<0.0001** | (N = 20) | (N = 12) | **< 0.0001** |
|  | (IQR = 216.8) | (IQR = 228.3) |  | (IQR = 327.0) | (IQR = 180.3) |  |
| **CD4+ T cell percentage** | 27.2 | 5.6 |  | 38.1 | 15.3 |  |
|  | (N = 17) | (N = 11) | **0.0001** | (N = 23) | (N = 14) | **0.0025** |
|  | (IQR = 18.4) | (IQR = 11.5) |  | (IQR = 32.6) | (IQR = 13.3) |  |
| **CD4/CD8 ratio** | 0.4 | 0.07 |  | 0.7 | 0.2 |  |
|  | (N = 16) | (N = 11) | **0.0002** | (N = 23) | (N = 14) | **0.0042** |
|  | (IQR = 0.4) | (IQR = 0.2) |  | (IQR = 0.9) | (IQR = 0.2) |  |
| **Viral Load (Units/µL)** | 49100 | 158500 |  | 33.5 | 75.5 |  |
|  | (N = 15) | (N = 10) | **0.027** | (N = 8) | (N = 6) | 0.40 |
|  | (IQR = 119770.0) | (IQR = 337643.0) |  | (IQR = 292.8) | (IQR = 11602.0) |  |

cART=Combination antiretroviral therapy; BMI=Body mass index.

**Supplementary table 2.** *SLC2A1* and *SLC2A1-AS1* SNP genotypes and the distribution of favorable and non-favorable CD4+Glut1+ T cell percentages in HIV-positive individuals.

| **HIV-positive treatment-naive** | | | | | | | | | | | **HIV+/cART** | | | | | | | | |
| --- | --- | --- | --- | --- | --- | --- | --- | --- | --- | --- | --- | --- | --- | --- | --- | --- | --- | --- | --- |
| **Total** | | | **Favorable progressors** | | | **Non-favorable progressors** | | | | | **Total** | | **Favorable responders** | | | **Non-favorable responders** | | | |
|  | **N** | **Hardy-weinberg**  **(P-value)** | **N**  **(%)** | | **CD4+Glut1+ T cell percentage (IQR)** | **N**  **(%)** | **CD4+Glut1+ T cell percentage (IQR)** | | | **Mann-Whitney test** | **N** | **Hardy-weinberg**  **(P-value)** | **N**  **(%)** | | **CD4+Glut1+ T cell percentage (IQR)** | **N**  **(%)** | | **CD4+Glut1+ T cell percentage (IQR)** | **Mann-Whitney test** |
| **rs1385129** | 28 |  |  |  |  |  | |  |  |  | 39 |  |  |  |  |  |  |  |  |
| **GG^a^** | 13 | 0.47 | 7  (53.8%) | | 16.0 (11.7) | 6  (46.2%) | 43.4 (11.0) | | | **0.0082** | 22 | 0.075 | 11  (50.0%) | | 10.8 (8.7) | 11  (50.0%) | | 16.8 (26.4) | 0.056 |
| **GA^b^** | 14 | (0.49) | 9  (64.3%) | | 18.0 (14.3) | 5  (35.7%) | 8.6 (31.9) | | | 0.67 | 15 | (0.78) | 12  (80.0%) | | 9.2 (5.7) | 3  (20.0%) | | 9.4 (7.0) | 0.82 |
| **AA^c^** | 1 |  | 1  (100%) | | 6.7 (-) | - | | - | | - | 2 |  | 2  (100%) | | 6.8 (-) | - | | - | - |
| **rs710218** | 29 |  |  |  |  |  | |  |  |  | 39 |  |  |  |  |  |  |  |  |
| **AA^a^** | 9 | 0.85 | 6  (66.7%) | | 15.8 (18.2) | 3  (33.3%) | 46.5 (1.9) | | | **0.024** | 20 | 2.15 | 11  (55.0%) | | 10.8 (8.9) | 9  (45.0%) | | 16.8 (27.6) | 0.095 |
| **AT^b^** | 12 | (0.36) | 6  (50.0%) | | 18.0 (13.4) | 6  (50.0%) | 18.1 (32.3) | | | 0.91 | 13 | (0.14) | 10  (76.9%) | | 10.3 (4.4) | 3  (23.1%) | | 14.5 (87.1) | 0.26 |
| **TT^c^** | 8 |  | 5  (71.4%) | | 19.0 (15.2) | 2  (28.6%) | 38.3 (-) | | | 0.095 | 6 |  | 4  (66.7%) | | 5.2 (3.3) | 2  (33.3%) | | 11.3 (-) | 0.27 |
| **rs1130214** | 29 |  |  |  |  |  | |  |  |  | 39 |  |  |  |  |  |  |  |  |
| **GG^a^** | 9 | 0.086 | 7 (77.8%) | | 15.0 (35.4) | 2  (22.2%) | 42.4 (-) | | | 0.25 | 14 | 0.27 | 11  (78.6%) | | 9.5 (5.5) | 3  (21.4%) | | 39.7 (28.7) | **0.011** |
| **GT^b^** | 15 | (0.77) | 7  (46.7%) | | 18.3 (19.0) | 8  (53.3%) | 31.4 (39.8) | | | 0.32 | 20 | (0.60) | 12  (60.0%) | | 10.4 (7.3) | 8  (40.0%) | | 15.7 (24.2) | 0.094 |
| **TT^c^** | 5 |  | 4  (80.0%) | | 21.2 (13.2 | 1  (20.0%) | 40.2 (-) | | | - | 5 |  | 2  (40.0%) | | 12.8 (-) | 3  (60.0%) | | 9.40 (8.4) | 0.80 |

cART=Combination antiretroviral therapy; IQR=Interquartile range.
